# Noise-compensated homotopic non-local regularized reconstruction for rapid retinal optical coherence tomography image acquisitions

**DOI:** 10.1186/1471-2342-14-37

**Published:** 2014-10-15

**Authors:** Chenyi Liu, Alexander Wong, Paul Fieguth, Kostadinka Bizheva, Hongxia Bie

**Affiliations:** 1 Department of Systems Design Engineering, University of Waterloo, Waterloo, Canada; 2 Department of Physics and Astronomy, University of Waterloo, Waterloo, Canada; 3 Department of Information and Communication Engineering, Beijing University of Posts and Telecommunications, Beijing, China

## Abstract

**Background:**

Optical coherence tomography (OCT) is a minimally invasive imaging technique, which utilizes the spatial and temporal coherence properties of optical waves backscattered from biological material. Recent advances in tunable lasers and infrared camera technologies have enabled an increase in the OCT imaging speed by a factor of more than 100, which is important for retinal imaging where we wish to study fast physiological processes in the biological tissue. However, the high scanning rate causes proportional decrease of the detector exposure time, resulting in a reduction of the system signal-to-noise ratio (SNR). One approach to improving the image quality of OCT tomograms acquired at high speed is to compensate for the noise component in the images without compromising the sharpness of the image details.

**Methods:**

In this study, we propose a novel reconstruction method for rapid OCT image acquisitions, based on a noise-compensated homotopic modified James-Stein non-local regularized optimization strategy. The performance of the algorithm was tested on a series of high resolution OCT images of the human retina acquired at different imaging rates.

**Results:**

Quantitative analysis was used to evaluate the performance of the algorithm using two state-of-art denoising strategies. Results demonstrate significant SNR improvements when using our proposed approach when compared to other approaches.

**Conclusions:**

A new reconstruction method based on a noise-compensated homotopic modified James-Stein non-local regularized optimization strategy was developed for the purpose of improving the quality of rapid OCT image acquisitions. Preliminary results show the proposed method shows considerable promise as a tool to improve the visualization and analysis of biological material using OCT.

## Background

Optical coherence tomography (OCT) [1] is a minimally invasive imaging technique, based on low-coherence interferometry, that utilizes the spatial and temporal coherence properties of optical waves backscattered from biological tissue. Given the high level of resolution (close to cellular) and non-invasiveness that can be achieved using OCT, a very promising application is in the in-vivo imaging of the retina for studying physiological processes as well as detecting retinal dystrophies in a clinical setting. Recent advances in swept source OCT (SS-OCT) and spectral domain OCT (SD-OCT) technology has resulted in image acquisition rates of hundreds to millions of A-scans per second [2,3]. The obvious advantages of the high data acquisition rates are the ability to image larger volumes of the imaged retina with sufficiently high pixel density in 3D, to allow for simultaneous visualizationof small and large scale morphological details in the retina, to track fast physiological processes in biological tissue, as well as to reduce the effect of motion artefacts resulting from natural motion in living biological tissue that can affect the quality of the retinal imaging.

One of the key challenges to rapid retinal OCT acquisitions is the increasing presence of noise as acquisition speed increases. Since OCT is based on the detection of partially coherent light, speckle noise is an inherent component of any OCT tomogram [4]. The presence of speckle results in a grainy appearance of the OCT images, which can blur the boundaries between features in the image with different structural or optical properties, or even obscure structural details of small size or low reflectivity. Moreover, the presence of speckle can affect negatively the performance of other image processing algorithms such as feature segmentation [5] and pattern recognition. Since speckle contains both information about the structure and optical properties of the imaged object and a noise component, different approaches were utilized in the past to suppress speckle noise and improve the image quality [4,6-8].

The presence of speckle noise is made worse by rapid OCT acquisitions, since the OCT signal-to-noise ratio (SNR) is directly proportional to the integration time of the signal detection and thus inversely proportional to the image acquisition rate [2,9,10], OCT imaging at the rate of hundreds of kHz or tens of MHz results in a significant drop in the image SNR. Therefore, morphological features in imaged biological tissue samples such as retinal tissue layers, small blood vessels, lipid deposits, etc, can be blurred or obscured by the presence of noise in unprocessed OCT images. Therefore, speckle noise reduction has drawn significant interest from the OCT community, since it can improve the image SNR and contrast, provide better visualization of morphological features in biological tissue that could be of clinical diagnostic value, as well as potentially improve the precision and overall performance of the other image post-processing algorithms such as layer segmentation, registration, cell detection, etc.

In general, these approached can be divided into two categories: instrumentation and software. Given the complexity, cost, and relatively limited gain in modifying the instrumentation to reduce the presence of noise, much attention has been focused on the software front. Previous studies on reducing speckle noise can be categorized into two groups: multi-frame averaging and digital image denoising approaches. The first strategy was mainly used for post-processing, where a sequence of B-scan images from a unique position are first captured, then registered and averaged to get a high SNR image [11,12]. Recently, a quantitative comparison of frame averaging approaches has been performed by Eichel et al. [13]. Some SD-OCT systems have a built-in registration and averaging system to do this post-processing progress automatically, such as Spectralis (Heidelberg Engineering, Heidelberg, Germany), which can help improve the image SNR directly.

Frame averaging has been proven to be simple and effective [11,14], however, it has two significant drawbacks: 

1. It results in overall increased imaging time since multiple B-scans must be acquired at the same location,

2. Precise image registration needs to be applied prior to averaging, which is time consuming and can lead to blurring in the frame-averaged tomogram if done incorrectly.

Another approach would be to use standard digital image denoising technologies to suppress speckle noise. An extensive comparison of standard digital denoising methods has been performed by Ozcan et al. [15]. Classic denoising algorithms often assume a priori parametric or non-parametric model for signal and noise, and operate on the reconstructed OCT tomogram in the spatial domain from a single acquisition to suppress noise. Some methods include adaptive non-linear filtering strategies [6,16-19], or wavelet filtering strategies [20-22]. More complicated wavelet thresholding denoising approaches such as dual tree complex wavelet transformation [23] and curvelets transformations [24], are able to generate satisfactory results in terms of improved image SNR with tolerable blurring. More recently, a weighted wavelet multiframe reconstruction algorithm was proposed [25] and used for preprocessing OCT for retinal layer segmentation, and a denoising algorithm was introduced based on a sparse representation dictionary approach [26,27]. However, all these denoising methods have the disadvantage that they have been designed to work only in the spatial domain, and therefore they do not take into account the inherent characteristics of the measured spectral signal from a SD-OCT system, which can lead to reduced performance in maintaining signal fidelity. A very interesting approach that was more recently taken was that is capable of not only reducing noise but also interpolate missing data using sparse representation dictionaries constructed from previously collected datasets [27].

In this paper, a noise-compensated homotopic modified James-Stein non-local reconstruction (NCHR) framework is introduced to improve the reconstruction of rapid retinal OCT image acquisitions, that can result in SNR and contrast-to-noise (CNR) improvements while preserving the sharpness and visibility of structural details in the reconstructed tomogram. The framework’s performance was tested on a series of human retinal OCT tomograms acquired in-vivo and was compared quantitatively with the performance of some of the most advanced published denoising approaches. It is important to note that, while it builds upon a homotopic reconstruction framework as with our previous work on sparse reconstruction [28], there are significant differences between the proposed work and our previous work, and as such highlights the main novel contributions of the proposed work: 

1. The work presented in [28] is designed for reconstructing OCT imagery from sparse spectral data acquired using compressed sensing, where a random sampling pattern is used to acquire incomplete measurements in the spectral domain. Since the acquisitions are made at regular scanning speed, the individual sparse measurements that were made have relatively higher SNR compared to that in this proposed work. Therefore, the goal of [28] is to reconstruct based on missing information, with the aim to allow for high resolution OCT imagery with limited camera pixels. However, the methodology in the proposed work is designed for reconstructing OCT imagery from rapidly acquired fully-sampled spectral data, where the scan speed is high and thus the amount of light captured at each scan is much lower than that in the sparse measurements case. Therefore, the goal of this work is to reconstruct based on fully-sampled but low-SNR acquisitions, with the aim to allow for rapid OCT imaging with higher effective SNR.

2. While both employ a homotopic minimization framework, the proposed work introduces a modified James-Stein non-local regularization strategy, while a conventional non-local regularization strategy is employed in [28]. As such, the proposed work is different and novel from an algorithmic standpoint as well relative to [28].

3. The proposed work incorporates a noise compensation strategy into the proposed homotopic modified James-Stein non-local regularized minimization framework to account for the noise characteristics of the underlying system.

The rest of the paper is organized as follows. First, the underlying methodology behind the proposed use of a homotopic modified James-Stein non-local regularization (NCHR) reconstruction framework for the reconstruction of rapid OCT tomograms is described in Section “Methods”. The experimental results using rapid in-vivo acquisitions of the human retina are presented and discussed in Section “Experiments”. Finally, conclusion are drawn and future work is discussed in Section“Conclusion”.

## Methods

The main principle behind the proposed approach is the introduction of a modified James-Stein non-local regularization strategy and noise compensation within a homotopic minimization framework.

**Figure 1:**
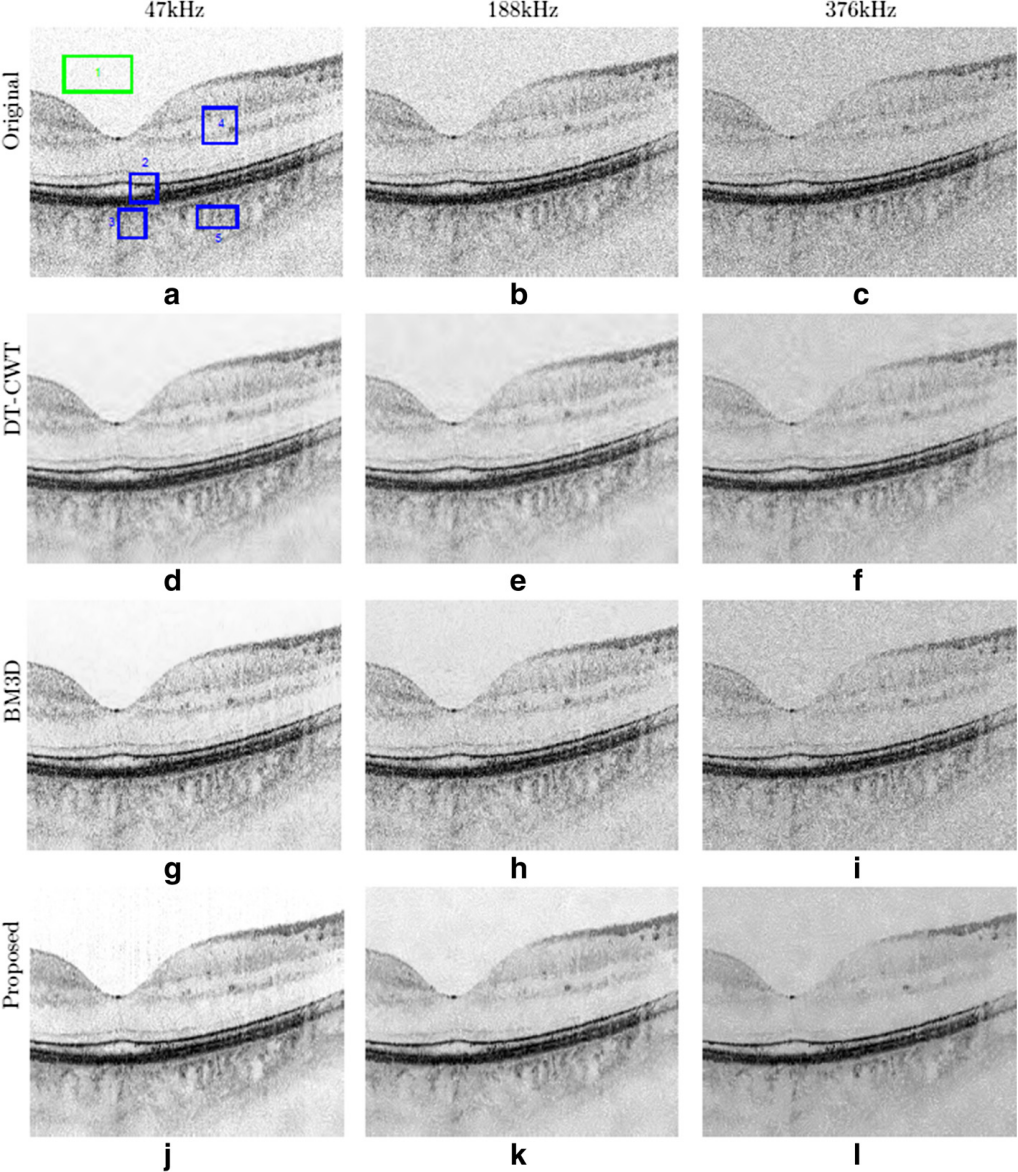
**Reconstruction results of three different methods.** 1**(a)**, **(b)**, **(c)** original images from 47 kHz, 188 kHz, and 376 kHz acquisitions; 1**(d)**, **(e)**, **(f)** reconstructed images from 47 kHz, 188 kHz, and 376 kHz acquisitions using DT-CWT; 1**(g)**, **(h)**, **(i)** reconstructed images from 47 kHz, 188 kHz, and 376 kHz acquisitions using BM3D; 1**(j)**, **(k)**, **(l)** reconstructed images from 47 kHz, 188 kHz, and 376 kHz acquisitions using NCHR. We can clearly observe the increase in noise level associated with higher scanning speeds. Our proposed method (NCHR) is able to suppress most of the noise while still preserving boundary and details, at the same time without losing any important structures. The green and blue line boxes in the original normal speed image mark regions which we used for perform quantitative comparison of all the processed images by different algorithms.

In SD-OCT, a broadband light source is split into reference and sample arms. The sample signal reflected back from the sample is recombined with the reference signal to produce broadband interference. The interference pattern is captured by a linear array CCD camera (each pixel capturing a different spectral frequency) and mapped into *k*-space. By applying a FFT, the *k*-space data is converted to the spatial domain, which allows for direct correlation of the spectral frequencies and the spatial locations within the imaged object where the optical imaging beam has been back-scattered. Let *S* be the set of sets in a discrete lattice *L* upon which an OCT image is defined in spatial domain, and *K* be a set of sites defined in a discrete lattice  upon which the same OCT image is defined in *k*-space domain. Denoting the measurements in spatial domain as *f*(*x*), and the measurements in *k*-space domain as *F*(*k*), the relationship between *f*(*x*) and *F*(*k*) is formulatedas

(1) $$f(x)=F^{-1}\left\{F(k)\right\}$$

where $F^{-1}$ denotes the inverse Fourier operator, and *F*(*k*) denotes measurements in *k*-space domain. However, solving the problem in this manner will take all the noise sampled in the *k*-space domain back to the spatial domain, thus causing detail blur as well as obscuring important structures, which we can see in Figure 1(a)(d)(g). Here we are promoting a different strategy for noise compensation under the proposed NCHR framework.

(2) $$\begin{array}{ll} \hat{f}(x) & =\underset{\sigma \to 0}{\lim}\underset{f(x)}{\text{arg}\;\text{min}}\;\rho (f(x),\sigma )\; \end{array}$$

such that

(3) $$\begin{array}{ll} \hat{F}(k) & =\left\{\begin{array}{ccccc} \hat{F}(k) & \text{if} & \left|\hat{F}(k)-F(k)\right| & < & \delta \\ F(k)+\delta & \text{if} & \hat{F}(k)-F(k) & > & \delta \\ F(k)-\delta & \text{if} & \hat{F}(k)-F(k) & < & -\delta \end{array}\right)\; \end{array}$$

where *ρ* is a modified James-Stein non-local regularization function, based on the work by James and Stein [29] and Wu et al. [30], which can be defined as

(4) $$\rho (\;f(x),\sigma )=\left[\underset{\text{x}\in \Omega }{\sum }\underset{i\in N(x)}{\sum }w(x,i,\sigma )\cdot {\left(R(x)-R(i)\right)}^{2}\right]$$

where *Ω* denotes the set of all the pixels in the image, *N*(*x*) stands for the neighborhood of the pixel *x*, and the term *w*(*x*,*i*,*σ*) is the modified James-Stein weight for the *i*-th neighborhood pixel, and we define *R*(*x*) as an operator that extracts a patch of a predetermined size from an image centered at *x*. The modified James-Stein weights, *w*(*x*,*i*,*σ*) are computed based on the amount of similarity of spatial neighborhoods extracted by operators *R*(*x*) and *R*(*i*),

(5) $$\begin{array}{c} w(x,i,\sigma )=\;\left(\;\alpha (x,i)\;-\;\left(\;1-\;\frac{(|N|-2)\sigma^{2}}{{∥f(x)-\hat{f}(x)∥}_{2}}\right)\;\;\right)\text{exp}\left\{\;-\frac{{\left(\hat{R}(x)-\hat{R}(i)\right)}^{2}}{2\sigma^{2}}\;\right\}\;, \end{array}$$

where |*N*| is the size of the neighborhood, and

(6) $$\alpha (x,i)=\left\{\begin{array}{ccccc} 1 & \text{if} & x & \neq & i\\ 0 & \text{if} & x & = & i \end{array}\right)$$

Based on Eq. 3, the proposed iterative strategy consists of two main steps (a detailed description is shown in Algorithm 1): first, modified James-Stein homotopic non-local regularization is enforced in the spatial domain, and then noise compensation is enforced via NCHR in the *k*-space domain to constrain the estimator based on the given frequency-domain information. This two-step process is repeated until convergence. To enforce modified James-Stein homotopic non-local regularization in an efficient manner, we employed an efficient steepest descent algorithm. Details regarding the implementation of this efficient steepest descent algorithm can be found in [31,32]. The minimization formulation in Eq. 3 reflects two objectives: 

1. Adjacent patches in a neighborhood should be similar;

2. The reconstructed signal should be in reasonable proximity to the measurements in the *k*-space domain.

In NCHR (Eq. 3), any coefficients that deviate from the original measurements beyond a certain tolerance *δ* are constrained back, coefficients that deviate from the original measurements beyond *δ* are constrained by lower and upper tolerance bounds. Therefore, the choice of *δ* becomes important as it will affect the noise compensation capabilities of the proposed framework. For example, choosing a *δ* that is too low would result in poor noise-compensation performance since the underlying noise would then be treated as important image content. However, choosing a *δ* that is too high would result in poor image reconstruction quality as the important image content would be treated as noise. Therefore, here, to choose the proper *δ* for the reconstruction framework, the noise floor of the imaging system operating at a particular speed is determined empirically and used to set *δ*. Different approaches for choosing *δ* to optimize noise compensation performance would be of interest as part of future investigation. 

## Experiments

### Data description

To evaluate the effectiveness of the proposed method we applied it to the reconstruction of a series of rapid human retinal OCT cross-sectional image acquisitions (Figure 1). The tomograms were acquired with a research grade, high-speed, UHROCT system [33], operating at 1060 nm wavelength, that utilizes a super-luminescent diode (*λ*-c = 1020 nm, *δλ* = 110 nm, *P*_*out*_ = 10 mW), a 47 kHz InGaAs linear array, and a 1024 pixel camera (SUI, Goodrich) interfaced with a high performance spectrometer. The UHROCT system provides 6 *μ*m resolution and 97 dB SNR for 1.5 mW of optical power incident on the cornea. Cross-sectional retinal tomograms were acquired from the foveal region of the retinas of healthy subjects using an imaging procedure that was approved by the University of Waterloo Office of Research Ethics. Written informed consent for participation in the study was obtained from the subjects. Each retinal tomogram was comprised of 1000 A-scans, each of 512 pixels. The raw OCT data was processed to generate images with SNR and CNR corresponding to the original data acquisition rate of 47 kHz, as well as corresponding to significantly higher scanning rates of 188 kHz (=4×47 kHz) and 376 kHz (=8×47 kHz) (simulated). In the implementation of NCHR, the parameters were set to *σ*_1_=0.3, and *λ*=0.7, and a patch size of 9×9 and neighborhood size of 21×21. The parameters used in the implementation of NCHR were found to provide strong results based on extensive empirical testing.

## Results and discussion

For comparison purposes, the proposed method (NCHR) was compared with two state-of-art denoising algorithms, DT-CWT [22] and BM3D [34], using the implementations provided by the authors of the respective works, with the determined noise levels at the different scan speeds used as inputs to the algorithms. To perform a comprehensive and systematic assessment of the reconstruction performance of the different methods, the signal-to-noise ratio (SNR) and the contrast-to-noise ratio (CNR) was computed for reconstructed data. These metrics are the same as those used in [4,6,7,35], and can be defined as follows:

(7) $$\text{SNR}=10{\text{log}}_{10}\left[\text{max}\left(A^{2}\right)/\sigma^{2}\right]$$

(8) $$\text{CNR}=\frac{1}{R}\left[\overset{R}{\underset{r=1}{\sum }}\frac{\left(\mu_{r}-\mu_{b}\right)}{\sqrt{\sigma_{r}^{2}+\sigma_{b}^{2}}}\right]$$

**Figure 2:**
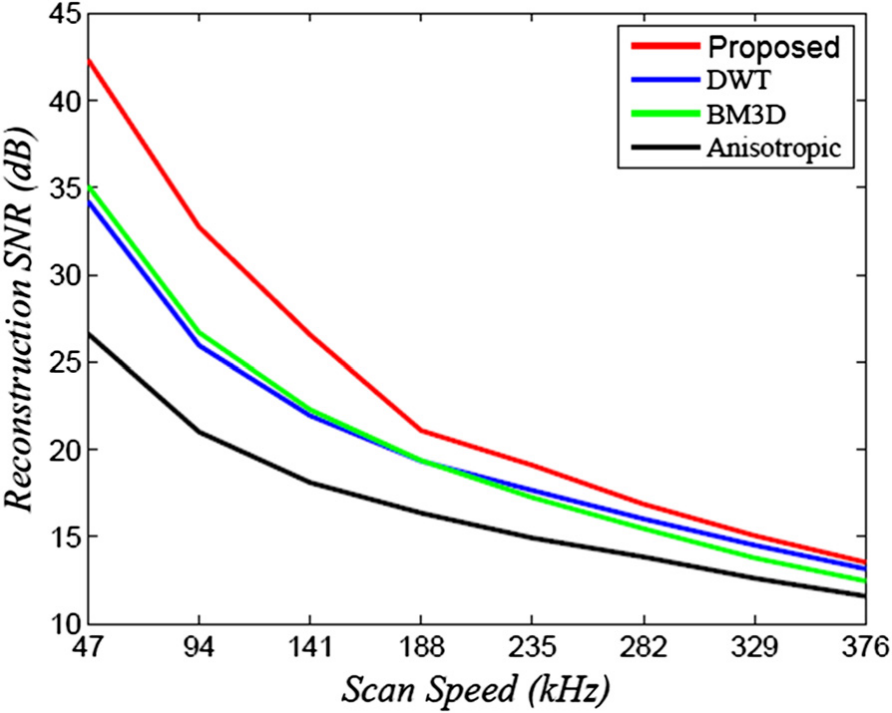
**SNR of three different reconstruction methods as a function of scan speed.** The proposed NCHR method produces reconstructed OCT data with higher SNR values for all scan speeds when compared to the other two methods.

**Figure 3:**
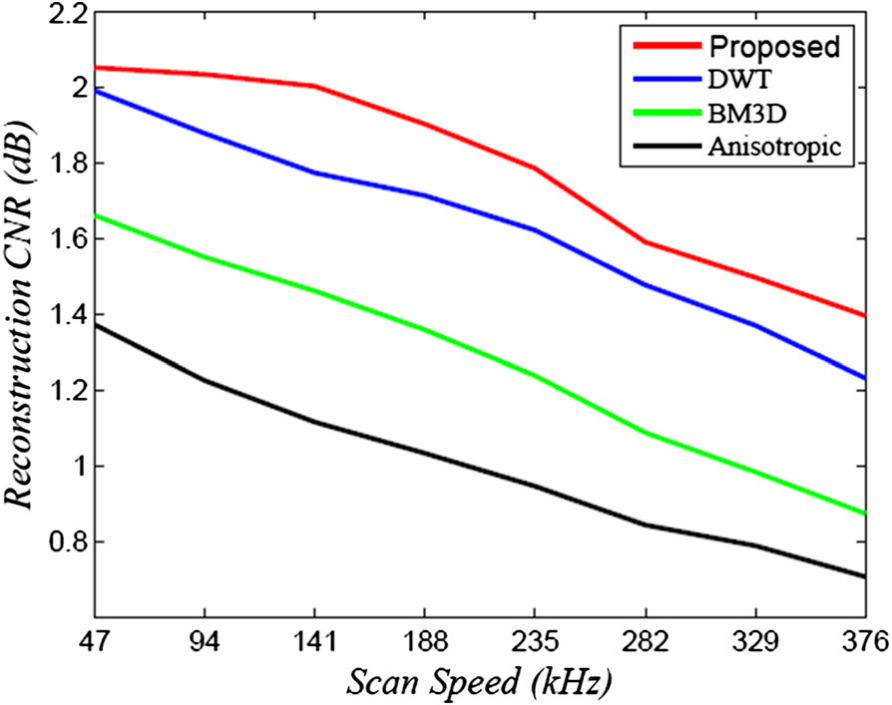
**CNR of three different reconstruction methods as a function of speed.** The proposed NCHR method produces reconstructed OCT data with higher CNR values at all scan speeds when compared to the other two methods.

**Figure 4:**
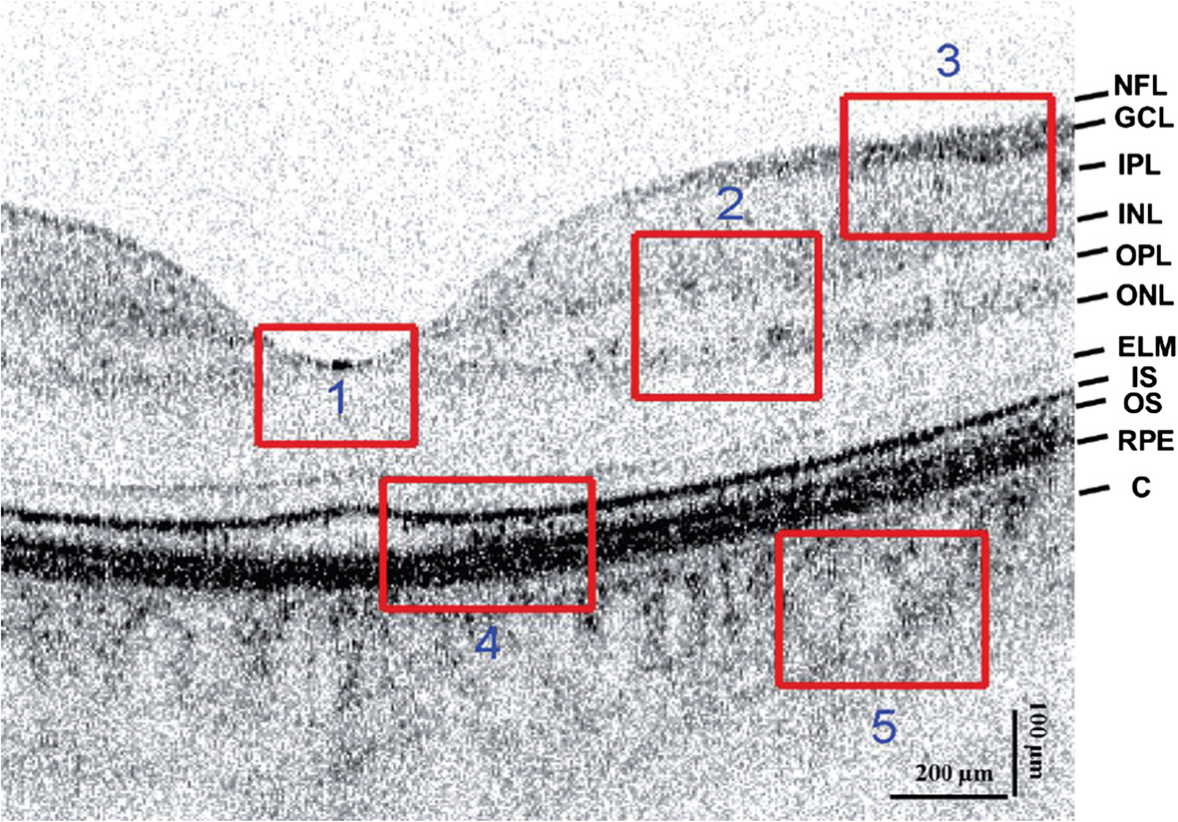
**UHROCT image of the human retina acquired near the fovea.** NFL denotes nerve fiber layer; GCL denotes ganglion cell layer; IPL denotes inner plexiform layer; INL denotes inner nuclear layer; OPL denotes outer plexiform layer; ONL denotes outer nuclear layer; ELM denotes external limiting membrane; IS denotes inner segment; OS denotes outer segment of the photoreceptor layer; RPE denotes retinal pigmented epithelium; C denotes choroid and S denotes sclera. Red line boxes mark regions of interest that were enlarged for more detail visual comparison in Figure 5.

**Figure 5:**
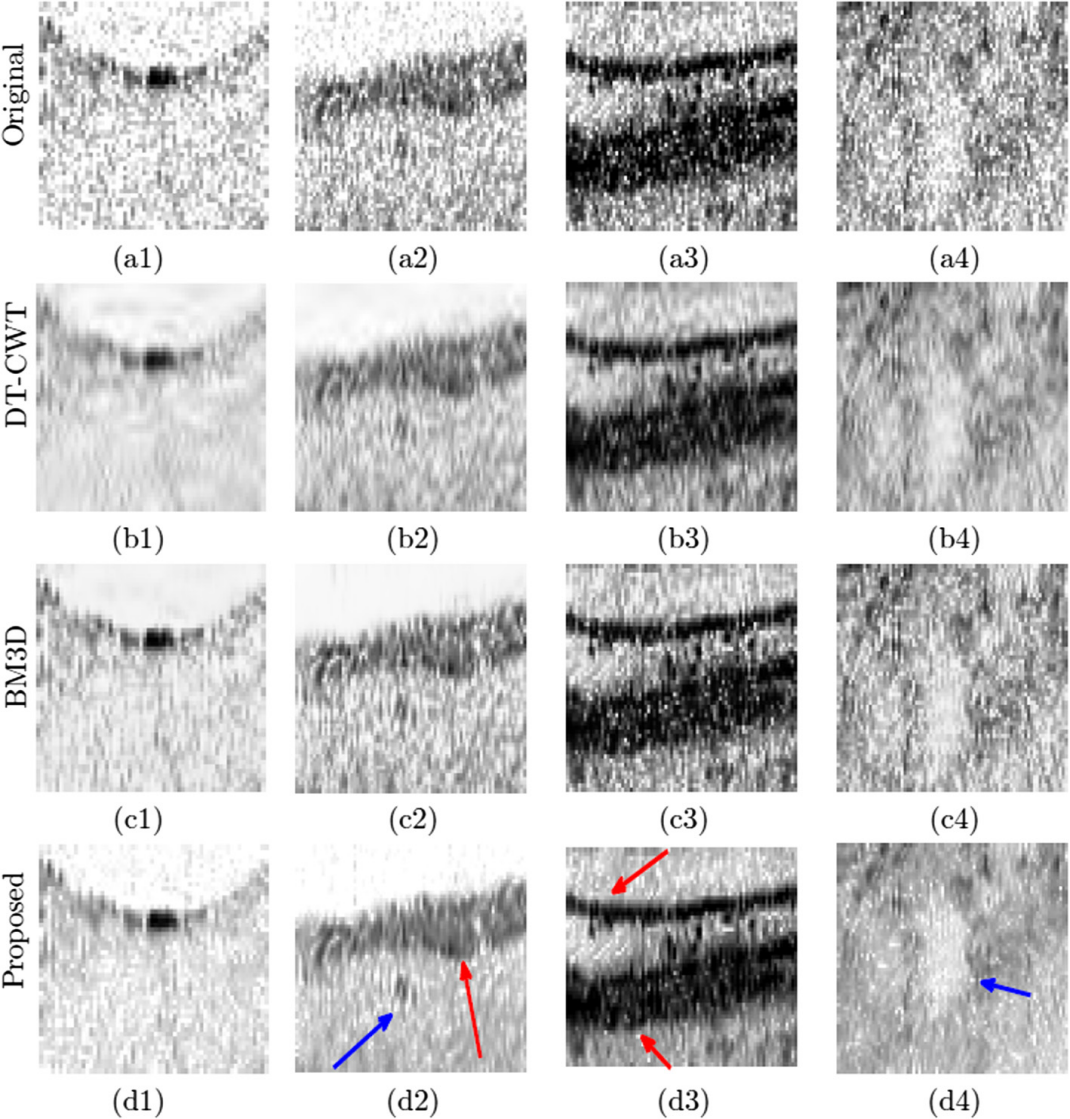
**Magnified view of the red rectangular regions shown in Figure**4**, and processed results of three different algorithms (DT-CWT, BM3D, Proposed).** 5**(a1)**, **(a2)**, **(a3)**, **(a4)** magnified regions from original image; 5**(b1)**, **(b2)**, **(b3)**, **(b4)** magnified regions from reconstructed image using DT-CWT; 5**(c1)**, **(c2)**, **(c3)**, **(c4)** magnified regions from reconstructed image using BM3D; 5**(d1)**, **(d2)**, **(d3)**, **(d4)** magnified regions from reconstructed image using NCHR; It can be observed that the proposed method (NCHR) results in improved important tissue contrast for the boundaries (see red arrows) and important features (see blue arrows), which can be very helpful for clinical and research purposes.

In the expression for SNR, *A* and *σ*^2^ represent the maximum magnitude of signal and variance of background noise region respectively. In terms of CNR, *μ*_*b*_ and $\sigma_{b}^{2}$ represent the mean and variance of the same background region as in SNR formulation, *μ*_*r*_ and $\sigma_{r}^{2}$ represent the mean and variance of the *r*^*t* *h*^ region of interest which includes the homogeneous regions.The SNR and CNR of the reconstructed OCT data sets is shown in Figures 2 and 3, as a function of the imaging speed. The proposed method resulted in significant performance improvements over the other two approaches of over 10 dB for normal speed to 5 dB for triple speed, and is consistently relatively higher for even more higher speed. Further more, the proposed method produced the best CNR compared with DT-CWT and BM3D, across all different speeds. It is important to note that beyond a scan speed of 144 kHz, there are no obvious advantages in terms of SNR of the proposed method when compared to DT-CWT and BM3D. As such, the optimal scan speed for gaining clear advantages from the proposed method is 144 kHz.Figure 1(a) shows the original image acquired with the maximum CCD data acquisition rate (47 kHz) image, while Figure 1(d), (g) and (j) show the reconstructed image using DT-CWT, BM3D, and our approach, respectively. Figure 1(b) corresponds to an image from 188 kHz acquisitions, which clearly shows a higher level of noise compared to Figure 1(a). The reconstructed images using NCHR are shown in Figure 1(e) and (f), separately. We can see very clearly that the DT-CWT approach (Figure 1(d)(e)(f)) leads to considerable blur, and the structure and detail information are hard to see. BM3D provides improved noise suppression compared to DT-CWT, but still has noticeable amount noise residual in the images (Figure 1(g)(h)(i)), which can blur important structural features and characteristics of the imaged retinal tissue such as the retinal layer. Finally, NCHR (Figure 1(j)(k)(l)) results relatively low residual noise and sharper boundaries of the imaging features such as the retinal layer boundaries, and does not appear to generate any noticeable artifacts.To quantify the performance of the tested algorithms, five regions of interest ROI were identified in the retinal tomogram of Figure 1 and marked with blue rectangles (Figure 4) and image metrics were applied to them. Those regions were chosen to contain different characteristics, layers, blood vessels, etc. Results from the performance of different algorithms are shown in different rows (Figure 5), while five enlarged regions were shown in different columns. Compared with the original noisy image, we can observe that all three methods successfully suppressed noise. DT-CWT tends to blur or smooth the whole images, while BM3D and our algorithm can improve the image contrast while still keep important tissues and structures. Figure 5(a2)-Figure 5(d2) show a region of the original image Figure 4 marked with redbox #2, containing sections of the NFL, GCL and IPL layer. The enlarged region from the original image(a2) shows the presence of the speckle reduces the overall image quality and blurs the boundary and obscure the important features as pointed out by red and blue arrows, respectively. DT-CWT blurs the whole image, BM3D and our proposed method give us better contrast, while our algorithm is also able to sharpen the important features and boundary compared with BM3D. Figure 5(a3)-Figure 5(d3) is a region of the original marked with red box #3, containing sections of the RPE layer(thick black line) and the IS/OS portion of the photoreceptor layer. Clearly our proposed method gives the sharpest boundary, as pointed by the red arrow, which has great potential in post-processing steps. For images Figure 5(a4)-Figure 5(d4), it is an important cell tissue in the human retina and it’s very important to distinguish the unique shape(marked with the blue arrow). DT-CWT suppressed the noise but also blurred the whole image, BM3D is able to suppress some noise, our algorithm is able to suppress noise while preserving the RPE layer. To summarize, our proposed method has the advantage of suppressed noise while preserving the sharpness and visibility of structural details in the retinal tomograms. In addition, compared to other state-of-art algorithms, our novel approach results in a significant improvement in image contrast.

Therefore, based on both quantitative SNR and CNR analysis as well as qualitative visual assessment, NCHR provided improved reconstruction performance and visual quality compared to all other algorithms. Finally, we performed a performance analysis between the modified James-Stein homotopic non-local regularization strategy and the conventional homotopic non-local regularization strategy within the context of the proposed reconstruction framework, and found that the modified James-Stein homotopic non-local regularization strategy to achieve an average SNR and CNR increase of 1.3 dB and 0.02 dB, respectively, across scan speeds compared to the conventional homotopic non-local regularization strategy.

## Conclusion

A novel noise-compensation approach based on homotopic, non-local regularization was presented for reconstructing images from rapid retinal OCT acquisitions. Results show that the proposed approach is able to achieve a significantly higher signal-to-noise ratio and better visual quality under all different scan speeds, thus illustrating the potential for obtaining high resolution images with lower equipment costs and reduced imaging times. Future work involves the study of the proposed approach for rapid corneal OCT acquisitions, which entails the investigation of different parameters and potentially modifications to the optimization framework.

## Competing interests

The authors declare that they have no competing interests.

## Authors’ contributions

Conceived and designed the NCHR methodology: CL, AW. Performed the experiments: CL, KB. Analyzed the data: CL, AW, PF, KB, HB. Wrote the paper: CL, AW, PF, KB, HB. All authors read and approved the final manuscript.

## Pre-publication history

The pre-publication history for this paper can be accessed here:

http://www.biomedcentral.com/1471-2342/14/37/prepub
